# Microfluidics-based point-of-care test for serodiagnosis of Lyme Disease

**DOI:** 10.1038/srep35069

**Published:** 2016-10-11

**Authors:** Samiksha Nayak, Archana Sridhara, Rita Melo, Luciana Richer, Natalie H. Chee, Jiyoon Kim, Vincent Linder, David Steinmiller, Samuel K. Sia, Maria Gomes-Solecki

**Affiliations:** 1 Department of Biomedical Engineering, Columbia University, 351 Engineering Terrace, 1210 Amsterdam Avenue, New York, NY 10027, USA; 2 Department of Microbiology, Immunology and Biochemistry, University of Tennessee Health Science Center, 858 Madison Ave, Memphis, TN, 38163, USA; 3 Immuno Technologies Inc, 20 South Dudley St, Memphis TN 38103, USA; 4 OPKO Diagnostics, LLC, 4 Constitution Way, Suite E, Woburn, MA, 01801, USA

## Abstract

Currently, diagnostic testing for Lyme disease is done by determination of the serologic responses to *Borrelia burgdorferi* antigens, with the exception of the early localized phase of disease where diagnosis must be done clinically. Here, we describe the use of microfluidics technology to develop a multiplexed rapid lab-on-a-chip point of care (POC) assay for the serologic diagnosis of human Lyme disease. Following ELISA screening of 12 candidate antigens, we tested 8 on a microfluidic diagnostic system, called mChip-Ld, using a set of 60 serological samples. The mChip-Ld test, which can be performed in 15 minutes at the point of care, showed promising performance for detection of antibodies to *B. burgdorferi* using the PPO triplex test (rP100 + PepVF + rOspC-K, AUC of 0.844) compared to a gold-standard reference of culture confirmed clinical samples. The performance is comparable to the commonly used C6 peptide by lab-based ELISA. In addition, the mChip-Ld test showed promising performance for early-stage diagnosis of the disease using the antigen OspC-K (sensitivity and specificity of 84% and 92%, respectively; AUC of 0.877). Overall, this study underscores the potential of using microfluidics to aid the diagnosis of Lyme disease at the point of care.

Lyme disease (LD) is widely distributed throughout temperate zones of the Northern Hemisphere^1^ but lacks a reliable point-of-care (POC) diagnostic test. Its prevalence is high and increasing. Newly diagnosed cases have doubled in the United States over the last decade^2^. The number of probable cases of Lyme disease has been revised upwards by 10 fold by the Centers for Disease Control and Prevention (CDC) to account for widespread under-reporting^3^: this number is now estimated at ~300 000 cases per year^4^.

Lyme disease is a progressive disease with a wide array of largely non-specific clinical manifestations gradually developing from early to late stage. Late disseminated infection is associated with permanent damage to the nervous and musculoskeletal systems^5^. *Erythema migrans* (EM) is the clinical sign of early infection (stage 1) in up to 80% of patients with classic Lyme disease^6^. Of the patients presenting with stage 1 LD, ~35% present with atypical rashes that are often misdiagnosed^7^, thereby putting a large group of patients at risk for developing late Lyme, antibiotic-refractory arthritis and/or post-treatment Lyme disease syndrome. In addition to *Borrelia burgdorferi* sensu stricto, the CDC recently reported the discovery of a new spirochete species (*Borrelia mayonii*) that causes LD in people in the upper Midwest^8^. The newly discovered Lyme causing *B. mayonii* is associated with additional symptoms not previously described for LD (nausea and vomiting) and with diffuse rashes rather than the classic Bull’s Eye of EM which further complicates clinical diagnosis of the disease^8^. About 15% of patients treated with the recommended 2–4 week course of antibiotics will have lingering symptoms of fatigue, pain or joint and muscle aches that can last more than 6 months. Between the population of patients that present at the clinic with atypical rashes^7^ and patients that are correctly diagnosed but go on to develop symptoms of late LD, a physician in an endemic area can be faced up with ~50% of patients at risk of developing late disease.

Prompt diagnosis and treatment is critical to prevent disease progression. Unlike most bacterial diseases that can be defined microbiologically by direct observation, LD is currently defined indirectly through serologic assays given that Lyme-causing *Borrelia* grows slowly (up to 6 weeks) in culture^9^. Current laboratory based serologic assays employ the C6 ELISA or a two-tier test comprised of C6, whole-cell or recombinant antigen ELISA followed by Western blot containing a number of *B. burgdorferi* antigens such as VlsE, p100, p66, p58, p45, p41, p39, p30/31, p28 and p18. The sensitivity of these assays varies between 35–56% for Early Stage I, 73–77% for Early Stage II and 96–100% for Late Stage III LD^10^,^11^,^12^,^13^,^14^. However, only 10 to 50% of patients with culture confirmed very early localized Lyme disease (EM rash < 7 days) presented a detectable antibody response using the sero-analysis technology tested^15^,^16^. A recent study found that the C6 ELISA can substitute for immunoblots in the two-tiered testing protocol for LD without a loss of sensitivity or specificity^17^. Thus, a rapid serodiagnostic assay which can reproduce the performance of the C6 ELISA would fill a significant void. Here, we describe how we used microfluidics technology to develop a quantitative multiplexed rapid lab-on-a-chip point of care (POC) assay for the serodiagnosis of human Lyme disease. Development of an assay or biomarkers that allow physicians to diagnose LD at the point of care enables prompt and proper treatment of patients.

## Methods

### Ethics Statement

The involvement of human subjects in the proposed studies falls under Exemption 4 as outlined under HHS regulations (45 CFR Part 46) and is not considered “clinical research” as defined by NIH. Blinded de-identified surplus serum samples from patients with signs and symptoms of Lyme disease enrolled in previous studies conducted by the Lyme Disease Center at Stony Brook University, reference Lyme disease panels from CDC, and reference healthy individuals from a commercial source were used. Informed consent was obtained from all patients enrolled in the studies that originated the samples. Use of these samples was approved under FWA00021769 by IntegReview, Inc. Ethical Review Board IRB #2. The methods were carried out in accordance with the relevant guidelines.

### Lyme Disease characterized human serum panel

#### Stony Brook Lyme Disease panel, n = 20 samples

Twenty samples from patients presenting at the clinic with signs and symptoms of Lyme disease, some of which were culture confirmed. These samples were used to do preliminary studies and improvement of testing parameters.

#### CDC LD panel, n = 40 samples

Thirty-five samples in this panel were collected from patients diagnosed with Lyme disease by experienced physicians in endemic areas (Northeast and upper-Midwest). Five of the samples included in the panel were obtained from healthy individuals from the same areas. An extensive set of information, ranging from detailed clinical symptoms at presentation to serologic data from ELISA and Western Blot for these patients is shown in Table S1. In addition, *B. burgdorferi* was cultured from 88% of the early Lyme cases and the remaining patients met a rigorous case definition for early disseminated or late Lyme disease and had a seropositive ELISA result. This panel as well as all information on its clinical characterization was kindly provided by Dr. Martin Schriefer from the NCID/CDC.

#### Cross-Reactive Human Sera Panel, n = 25 samples

Sera from healthy patients from a non-endemic area (Golden West Bio, Tennessee) was used to do specificity studies.

### Protein Purification

OspA (outer surface lipoprotein A), OspB (outer surface lipoprotein B), OspC-K (outer surface lipoprotein C type K) and OspC-B (outer surface lipoprotein C type B) were purified in our laboratory as follows. Recombinant *E. coli* clones were grown in Tryptone Broth Yeast (TBY) medium supplemented with 50 μg/ml Kanamycin (Kn) at 37 °C, shaking at 225 rpm, until it reached an OD_600_ of 0.8. The expression of 6xHis tagged recombinant proteins was induced by adding 1 mM IPTG (isopropyl-β-d-thiogalactopyranoside) to the cells followed by incubation at 37 °C for 3 h. The cells were harvested by centrifugation at 4000 × g for 10 min at 4 °C. The proteins were purified by affinity chromatography using the Ni-NTA Purification System (Invitrogen) following the manufacturer instructions. Protein concentration was determined by the Bradford protein assay (Bio-Rad, Hercules, CA, USA), and was stored at −80 °C. Pure recombinant proteins (crude extract) were analyzed on a 10% denaturing polyacrylamide gel and electrotransferred to a polyvinyldene difluoride membrane (PVDF, Millipore, Billerica, MA) for analysis with antigen-specific-polyclonal mouse antibody. Purified recombinant proteins from *B. burgdorferi* such as flaB (flagellin B, p41), p100 (membrane lipoprotein p100/p93), BmpA (laminin binding protein A, p39), DbpA (decorin binding protein A), DbpB (decorin binding protein B) were purchased from ProSpec (Rehovot, Israel); VlsE (Variable Major Protein like sequence E, surface exposed lipoprotein) was purchased from My BioSource (San Diego, CA).

### Peptides

The following peptides were used: pepBBK07 (kindly provided by Dr. Utpal Pal, U. of Maryland) and PepVF (synthesized at GenScript, Piscataway, NJ). PepVF design: we modified a peptide based in the core sequence from the full length 25-residue IR6 from *B. burgdorferi* B31^18^ by adding a 13 amino-acid sequence from FlaB. Crude extract of PepVF was synthetized by GenScript.

### Serological immune responses

Human serum was tested for the presence of IgG against purified recombinant proteins and peptides by indirect ELISA. A list of antigens is described in Table S6. Antigens were coated on flat-bottom ELISA plates at 2 μg/ml (Nunc MaxiSorp™, ThermoFisher) and indirect ELISA was performed using human serum (1:100). Goat anti-human IgG (1:50,000) horseradish peroxidase-conjugated antibody (Jackson Immunoresearch, USA) was used as secondary antibody. Four healthy samples from the CDC panel were used (3 standard deviation above the mean) to determine the cutoff of the assay in Method 1; one healthy sample was positive against all recombinant *B. burgdorferi* proteins and was excluded from this study. Method 2 cutoff was determined by ROC curve analysis and choosing a point that maximized sensitivity and specificity.

### Point-of-care immunoassay

**Figure 1:**
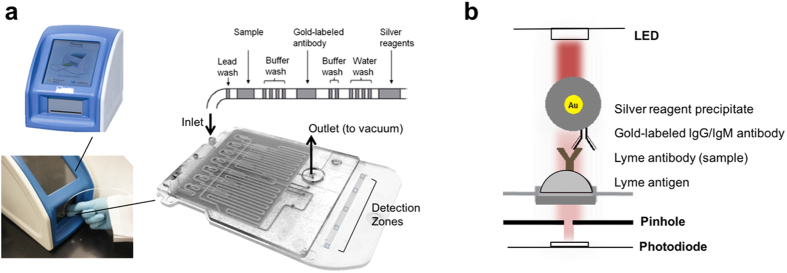
Overview of the mChip-Ld Device. (**a**) The plastic microfluidic cassette is inserted into a benchtop analyzer (Claros1 Analyzer, OPKO) which is used to power fluid flow, control temperature setpoints and detect signals. Sera samples as well as all washes, gold-labeled secondary antibodies and silver amplification reagents are pre-loaded and delivered automatically in sequence, passing over the five detection zones of the microfluidic channel. Pressure driven flow is achieved through attachment of a vacuum (simple syringe or benchtop analyzer) to the microfluidic cassette outlet. (**b**) Schematic of the biochemical and optical set up. Lyme antigens are adsorbed to the surface of the plastic microfluidic cassette. Sequential binding of the sample antibodies, gold-labeled detection antibodies and silver amplification reagents results in a visible signal that can be quantified as the optical density of the detection zone. Light emitted from a LED above the detection zone is collected by a photodiode. The presence of silver development, which absorbs incident light, reduces light sensed by the photodiode.

Injection molded plastic microfluidic cassettes were functionalized by direct adsorption of antigen candidates (purified recombinant proteins and peptides of *B. burgdorferi*) at the following concentrations: 100 μg mL^−1^ PepVF, 20 μg mL^−1^ rOspC-K, 30 μg mL^−1^ rOspC-B, 1 μg mL^−1^ rVlsE, 200 μg mL^−1^ rP41, 60 μg mL^−1^ rP100, 30 μg mL^−1^ rDbpB, 100 μg mL^−1^ rBmpA, and 20 μg mL^−1^ rDbpA. Avidin-biotin conjugation was used for pepBBK07 functionalization: 50 μg mL^−1^ streptavidin followed by 60 μg mL^−1^ pepBBK07. Functionalized cassettes included an internal negative control zone, spotted with no antigen, an internal positive control zone, spotted with 20 μg mL^−1^ rabbit anti-goat IgG antibody (Life Technologies) and multiplexed combination of three target antigen zones. All zones were treated for 1 hour with 1% BSA-0.05% Tween-20 in PBS for blocking and stabilizing. Further details on cassette preparation can be found in previous studies^19^,^20^,^21^,^22^. To run the assay, polyethylene tubing (inner diameter: 0.86 mm, Zeus) was pre-loaded with all reagents and connected to the cassette inlet for delivery, in a method previously described^19^,^20^,^23^; initial wash of 2 μL of 0.05% Tween-20 in PBS, 30 μL of serum sample (10X dilution in 1% BSA), four 2 μL 0.05% Tween-PBS washes, 14.5 μL secondary gold-conjugated anti-hIgG (1.06 ug/mL) and anti-hIgM (0.54 ug/ml) antibodies (OPKO Diagnostics) in 3% BSA-0.2% Tween-20 in PBS, followed by two 2 μL 0.05% Tween-PBS washes and four 2 μL water washes, each separated by air spaces (Fig. 1a). As in previous work, these steps take about 10 minutes to flow (which includes all the binding steps of the immunoassay)^21^. Silver nitrate and reducing agents (OPKO Diagnostics) were subsequently drawn through the cassette. An initial intensity reading (*I*_0_) was taken immediately after silver entered the channel, and another intensity reading (*I*) was taken after 4.5 minutes of silver development. All experiments were read on a bench-top analyzer (OPKO Diagnostics) (Fig. 1a). Optical density was calculated as:


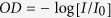


### Statistical analysis

In one method, receiver operating characteristic (ROC) analysis was performed to determine cutoff values and assess sensitivity and specificity. In another method, cutoff values were established at 3STDEV above the average of four negative control samples. 95% Confidence Intervals (95% CI) were calculated. Calculations were performed using Graphpad Prism. For markers containing multiple antigens (e.g. the three antigens multiplexed tests), we used a 1:1:1 ratio of weighting constants to add up the signals, and then used ROC analysis to compare AUCs with a new cutoff, similar to evaluation of different permutations of biomarkers as demonstrated in other works evaluating pooled set of markers^24^,^25^.

## Results

### Point-of-care device

The POC device, which we call mChip-Ld, consists of a signal detection device and a disposable, injection molded plastic cassette on which all the biochemical steps of the immunoassay are carried out (Fig. 1). Lyme-specific antibodies in a patient blood sample bind to antigens immobilized on the surface of the microfluidic cassette. Subsequent automated reagent delivery of all washes, secondary antibodies and silver amplification reagents results in silver ion reduction on gold nanoparticles attached to cassette surface. The detection mechanism, as previously described^19^, consists of paired light-emitting diodes (LEDs) and photodetectors aligned directly with the microfluidic test zones (Fig. 1a). When inserted into the analyzer, each microfluidic test zone of the cassette is sandwiched between a red LED aligned above with a 1 mm pinhole and red-sensitive photodiode aligned directly below each test zone. Silver development on the cassette results in a proportional decrease in the light sensed by the photodiode and can be quantified by optical density values (Fig. 1b). Assay time to result is approximately 15 minutes.

### Screening of candidate antigens

**Figure 2:**
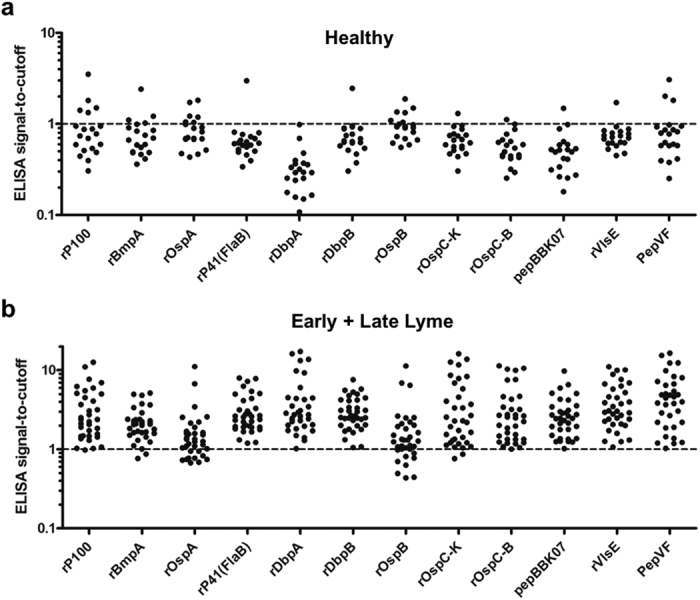
ELISA screening of candidate antigens for Lyme diagnosis. Signal to cutoff plots showing specificity and sensitivity of candidate antigens using IgG ELISA compared to reference testing with clinical evaluation and cultures. (**a**) 25 sera samples from healthy individuals in a non-endemic area and (**b**) 35 sera samples clinically characterized as positive by the CDC for Lyme Disease were used to test specificity and sensitivity of candidate antigens of rP100, rBmpA, rOspA, rP41(FlaB), rDbpA, rDbpB, rOspB, rOspC-K, rOspC-B, pepBBK07, rVlsE and PepVF.

In order to develop multiplexed panel designs for the POC test, we first screened candidate antigens using conventional ELISA. We examined 12 candidate recombinant antigens: rP100, rBmpA, rP41 (FlaB), rDbpA, rDbpB, rOspA, rOspB, rOspC-K, rOspC-B, rVlsE, pepBBK07, and PepVF (Fig. 2, Table S6). We used a panel of Lyme positive samples (n = 35) characterized by the CDC and healthy samples taken from non-endemic areas (n = 25). Two cutoff methods were evaluated in the screening of candidate markers on ELISA. In Method 1, four healthy samples from the same area of collection as the Lyme positive samples were tested. Cutoff for each antigen was determined as 3 standard deviations above the mean signal of these four samples, thus prioritizing a high specificity test. In Method 2, receiver-operator curve (ROC) analysis was used to select a cutoff maximizing sensitivity and specificity looking at area under the curve (AUC), prioritizing a high sensitivity test. The full table of results is shown in Table S2. For the intended application of designing a high sensitivity test for screening decisions, Method 2 was chosen for subsequent analysis.

When testing the full panel of Lyme samples by ELISA, covering both Early and Late Stage Lyme, rP41, rDbpA, rDbpB, rOspC-B, rVlsE, pepBBK07 and PepVF showed the highest sensitivities (100% sensitivity) and specificities (>90%) (Fig. 2, Table S3). The following proteins were highly sensitive as diagnostic candidates using Early Lyme test samples (>95% sensitivity): rP100, rP41, rDbpA, rDbpB, rOspC-K, rOspC-B, rVlsE, PepBBK07 and pepVF (Table S3A). Using Late Lyme samples we identified the following antigens (100% sensitivity): rP100, rBmpA, rP41, rDbpA, rDbpB, rOspC-B, rVlsE, pepBBK07 and PepVF (Table S3B). The least cross-reactive antigens (>90% specificity) tested against serum samples from healthy individuals were: rP41, rDbpA, rDbpB, rOspC-K, rOspC-B, rVlsE and pepBBK07 (Fig. 2a). rOspA with sensitivity of 71.4% (95% CI: 54–85%), specificity of 70.0% (95% CI: 46–88%) and AUC of 0.784, as well as rOspB with sensitivity of 74.3% (95% CI: 57–87%) specificity of 70% (95% CI: 46–88%) and AUC of 0.714 showed the poorest performance compared to other antigens and were eliminated as candidates for POC testing (Table S3). By deconstructing diagnostic performance with the full sample panel into Early Lyme (Table S3A) and Late Lyme (Table S3B), we also illustrate the potential of candidate antigens for disease staging as well as diagnosis.

**Figure 3:**
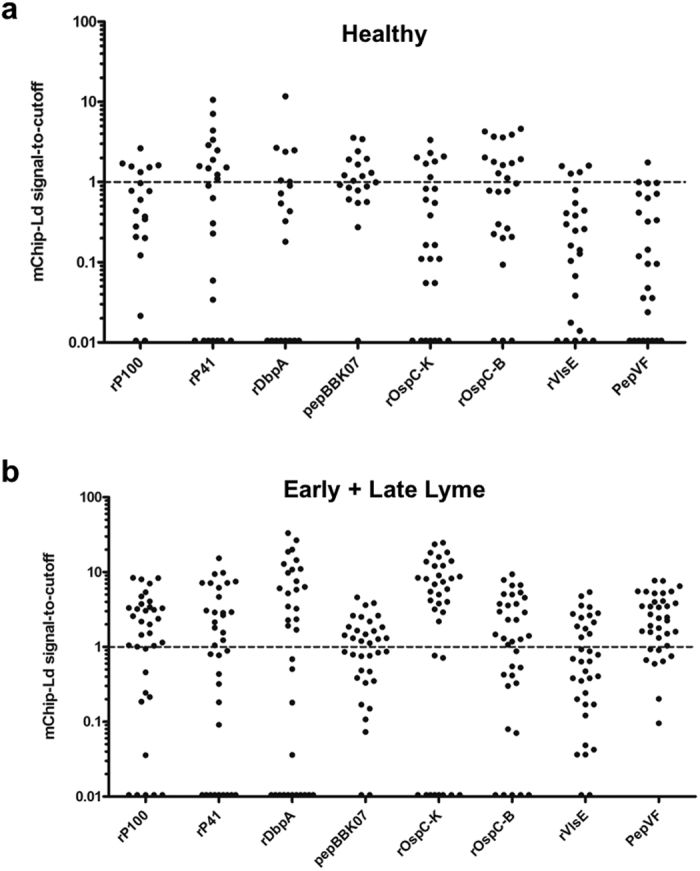
Preliminary screening of candidate antigens with mChip-Ld. Signal to cutoff plots showing specificity and sensitivity of candidate antigens on the mChip-Ld platform (IgM, IgG). Recombinant antigens and peptides (rP100, rP41(FlaB), rDbpA, pepBBK07, rOSpC-K, rOspC-B, rVlsE and PepVF) were chosen for testing from the ELISA screening study. (**a**) 25 sera samples from healthy individuals in a non-endemic area and (**b**) 35 sera samples clinically characterized as positive by the CDC for Lyme Disease were used to test specificity and sensitivity of candidate antigens.

To characterize sensitivity and specificity of these antigens for application in Lyme diagnosis using the rapid microfluidics format, we performed a preliminary screening test. Due to the large number of antigens being screened (ten), we did not optimize this test as much as some previous studies on the mChip system^19^,^20^,^21^,^22^. For example, we did not optimize the conjugation chemistry beyond physiosorption, concentration of coating protein, and blocking conditions. Instead, we looked for the markers that produced the best relative performance within the mChip-Ld data set. Twenty patient samples were used to evaluate various permutations of multiplexed markers with surface conditions suitable for a panel (i.e. balancing conditions for optimal signal-to-noise ratios for most markers on a panel, not necessarily the highest signal-to-noise ratios that can be recorded on the mChip-Ld platform for an individual marker). Next we screened the immunoassay potential of individual candidate antigens (rP100, rP41/FlaB, rDbpA, rOspC-K, rOspC-B, rVlsE, pepBBK07 and PepVF) on the mChip-Ld POC platform against the same Lyme positive and healthy sample panels used in ELISA screening experiments (Fig. 3, Tables S4 and S5). rOspC-K and PepVF were highly sensitive (>80% sensitivity) as diagnostic candidates using Early Lyme test samples (Table S5A). Using Late Lyme samples, we identified the following antigens (>70% sensitivity): rP100, pepBBK07, rVlsE, rOspC-B and PepVF, with the latter two markers having >90% sensitivity (Table S5B). The least cross-reactive antigens (>80% specificity) tested against serum samples from healthy individuals were: rVlsE, rOspC-K and PepVF (Fig. 3a). A breakdown of Early and Late Lyme classification by candidate antigen on the POC platform show that OspCs both skew towards ‘positive” disease classification for Early Lyme and ‘negative’ for Late Lyme, suggesting potential for discrimination between disease stages (Table S5).

### Comparison of ELISA with the multiplexed microfluidic chip test (mCHIP-Ld)

**Figure 4:**
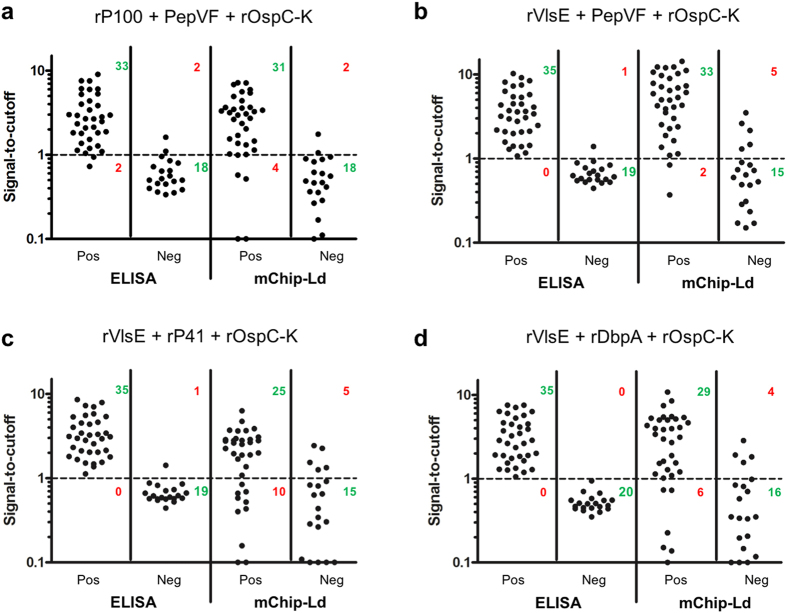
Comparison of ELISA and an initial version of multiplexed microfluidic chip platform (mChip-Ld). A vertical scatterplot showing signal-to-cutoff ratios of samples positive (Pos) or negative (Neg) for Lyme Disease, as determined by gold standard tests of clinical confirmation and culture tests, on ELISA and mChip platforms using a multiplexed combination of (**a**) rP100, PepVF and rOspC-K; (**b**) rVlsE, PepVF and rOspC-K; (**c**) rVlsE, rP41 and rOspC-K, and (**d**) rVlsE, rDbpA and rOspC-K.

Next, we analyzed four potential multiplex panel designs: (1) rP100 + PepVF + rOspC-K; (2) rVlsE + PepVF + rOspC-K; (3) rVlsE + rP41 + rOspC-K, and (4) rVlsE + rDbpA + rOspC-K on both ELISA and POC platforms compared to gold standard reference tests of clinical classification and culture tests (Fig. 4, Table 1). A simple multivariate model was used to combine the signals for each marker and develop a new cutoff in order to classify samples as positive or negative in these multiplexed tests. rP100 + PepVF + rOspC-K, had 94.3% sensitivity 90% specificity on ELISA compared to 88.5% sensitivity, 90% specificity (AUC: 0.844) on the mChip-Ld system (Fig. 4a, Table 1). The remaining combinations of: (2) rVlsE + PepVF + rOspC-K, (3) rVlsE + rP41 + rOspC-K and (4) rVlsE + rDbpA + rOspC-K all had 100% sensitivity, and >95% specificity on ELISA. On the mChip-Ld system, combination (2) of rVlsE + PepVF + rOspC-K yielded the highest sensitivity of 94.3%. with 75% specificity (AUC: 0.932) (Fig. 4b, Table 1). The triplexed panel rP100 + PepVF + rOspC-K (Fig. 4a) showed comparable performance on both the ELISA and POC plaforms when compared to clinical classification and culture tests. rVlsE + PepVF + rOspC-K (Fig. 4b) had higher sensitivity than rP100 + PepVF + rOspC-K on the POC platform, however with a tradeoff of lower specificity with the cutoff chosen here.

**Figure 5:**
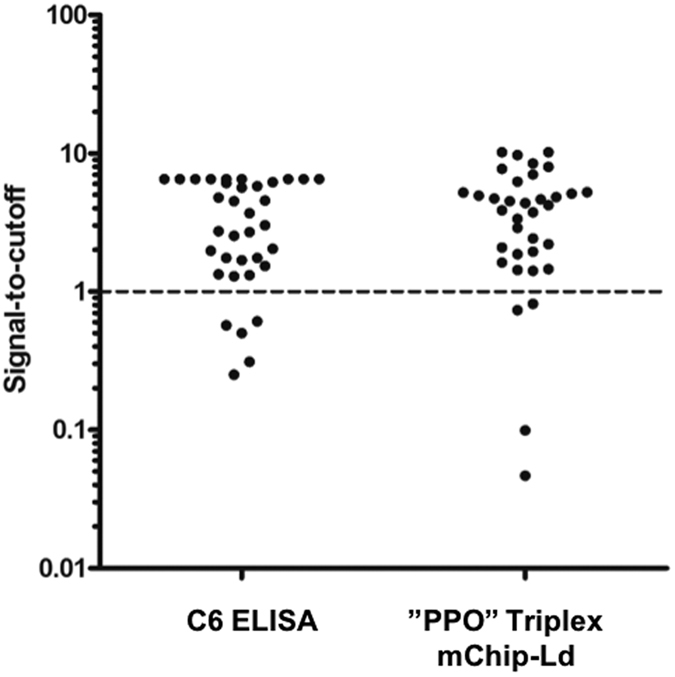
Comparison of C6 ELISA Lyme test with “PPO” triplexed mChip-Ld test. Vertical scatter plots showing samples previously validated to be positive for Lyme disease (Early and Late Stage) as tested on the C6 ELISA test (left) and multiplexed mChip device (right). The multiplexed mChip test used a combined signal of three proteins (rP100, PepVF and rOspC-K), and produced results comparable to the single-antigen C6 ELISA.

We compared the sensitivity of the current standard C6 ELISA to a multiplexed POC test consisting of rP100 + PepVF + rOspC-K (Fig. 5). The multiplexed POC test achieves comparable results to the C6 ELISA in identifying Lyme positive samples (88.5% mCHIP-Ld vs 85.7% C6 sensitivity).

## Discussion

Here, we report the development of a microfluidics based rapid assay for the serodiagnosis of Lyme disease with a performance comparable to the C6 ELISA with regard to sensitivity and specificity. The material cost of each cassette is about $1.50^20^,^21^. The assay can be done by minimally trained personnel in 15 minutes.

Previously we have demonstrated a dual HIV and syphilis immunoassay with a low-cost smartphone accessory, or “dongle” for use in resource-limited settings^20^,^21^. Here, we leverage the simple and low cost optics required for signal detection with a proprietary analyzer developed by OPKO Diagnostics, that can be packaged as a benchtop instrument or battery-operated hand held unit, with wireless, wired or printer output capabilities. Fluid handling, temperature control and signal detection modules are integrated and automated into a single device. The detection mechanism, as previously described^19^, consists of paired light-emitting diodes (LEDs) and photodetectors aligned directly with the microfluidic test zones (Fig. 1).

All the biomarkers tested have been shown to detect antibodies in blood from Lyme disease patients. Most antigens (rVlsE, rP93/100, rBmpA/P39, rFlaB/P41, rDbpA/P18, rDbpB/P18, rOspC/P23) are currently included in Western blot or line blot assays^26^,^27^. We found that two types of OspC (type K and type B) might be ideal to identify early Lyme disease samples^28^. OspA and OspB were identified as potential good candidates to discriminate late Lyme^29^. BBK07 immunodominant peptides were defined as good serodiagnostic markers for Lyme disease^30^,^31^. In addition, the C6 peptide ELISA was widely adopted for diagnosis of Lyme disease^10^,^32^,^33^,^34^. We modified a peptide based in the core sequence from C6 from *B. burgdorferi* B31^18^ and added a 13-aminoacid sequence from FlaB (pepVF). In the studies reported here, ELISA screening (Fig. 2) showed six recombinant proteins, p100, p41, DbpA, OspC-K, OspC-B, VlsE and two peptides pepBBK07 and pepVF, were promising antigens for diagnosis of Lyme disease. rOspA and rOspB showed the poorest sensitivity and specificity performance compared to other antigens and were eliminated as antigens for point of care.

mChip has comparable performance to ELISA but is faster and can be implemented at the point of care. Further, we can separate antigens per zone of detection, which allows the physician to make a comprehensive antigen-based diagnostic decision. Our ELISA results identified the eight antigen candidates for the microfluidics screen (Fig. 3). These studies led to the further elimination of two antigenic candidates, OspC-B and pepBBK07 due to high cross-reactivity with healthy samples. At this point we compared 4 combinations of antigens in multiplexed microfluidics and ELISA formats, rP100+PepVF+rOspC-K, rVlsE+PepVF+rOspC-K, rVlsE+rP41+rOspC-K and rVlsE+rDbpA+rOspC-K (Fig. 4). On ELISA our best combination was rVlsE+rDbpA+rOspC-K which detected accurately 35 LD positive samples and 20 negative healthy samples (100% sensitivity and specificity). On the microfluidics format, the best combination of antigens was the only one that did not contain rVlsE (rP100+PepVF+rOspC-K), which detected 31/35 positive LD samples and 18/20 negative healthy samples (88.57% sensitivity, 90% specificity, AUC of 0.844). VlsE underperformed in the microfluidics format as it produced higher cross-reactivity with healthy samples. In these studies, we did not optimize the conjugation chemistry beyond physiosorption for most antigens, and also did not optimize concentration of coating protein and blocking conditions. With additional clinical data sets, a more detailed, powered analysis for multiplexing can be undertaken, to refine coefficients in this model^24^,^35^. Further, we did not perform advanced assay development testing to identify positive samples from a blinded mixture of healthy and LD samples. Future optimization of this assay would include those studies. Another limitation of the POC assay, which can be generalized to all other serologic assays, is that it does not detect antibodies which may not be present in serum in the first two weeks post infection. In addition, future steps include optimization of assay parameters (such as anti-coagulation reagents and washing conditions) to work with whole-blood samples, as performed previously^20^,^21^. Also, prior work shows an application for our POC system in a Sub-Saharan setting where temperature and humidity can be significant factors; here, we anticipate primary usage of the Lyme POC for use in doctor’s offices and primary-care settings in the U.S where refrigeration is available for shipping and storage, and where environmental conditions are typically controlled.

We compared our best mChip-Ld candidate (rP100+PepVF+OspC-K) to the C6 ELISA which is generally used as a first tier assay for serodiagnosis of Lyme disease (Fig. 5, Supplementary Table 1) and we found that the sensitivity for overall diagnosis of Lyme is just as high for microfluidic test as for the C6 ELISA test. More than one-third of the Lyme-positive samples, covering both Early and Late Stage Lyme, were classified as “Negative” by Western Blot, though classified as “Positive” on C6 ELISA (Supplementary Table 1). In addition, the OspC-K antigen in a microfluidic format shows promising results as detection of early-stage LD.

One of the antigens used in our lead microfluidics assay, PepVF, is based on the 26-mer invariable region (IR(6)) of the variable surface antigen of *B. burgdorferi* (VlsE). This antigen is conserved among European pathogenic genospecies^36^ and it was reported recently that *B. mayonii* infection was identified by C6 ELISA^8^. Thus, we speculate that a rapid detection assay based on PepVF, as described in this study, should identify *B. bugdorferi* sensu lato infections.

Diagnostic testing for Lyme disease is traditionally achieved by determination of the serologic responses to *B. burgdoferi* sensu lato, with the exception of the very early localized phase of disease (EM < 10 days), in which the diagnosis must be done clinically due to the recognized lack of antibody availabe for detection by serologic assays^37^. In this study, we show that we can detect culture confirmed clinically characterized LD using an assay suitable for use at the point of care. The versatile nature of the microfluidcs platform allows us to explore development of a stand-alone assay for the future rapid diagnosis of Lyme disease that provides the physician with information on reaction to individual antigens.

## Additional Information

**How to cite this article**: Nayak, S. *et al.* Microfluidics-based point-of-care test for serodiagnosis of Lyme Disease. *Sci. Rep.* **6**, 35069; doi: 10.1038/srep35069 (2016).

## Supplementary Material

Supplementary Information

## Figures and Tables

**Table 1:** Comparison of ELISA and point-of-care (POC) multiplexed tests.

	Sensitivity (%)	CI (95%)	Specificity (%)	CI (95%)	AUC
ELISA: Early + Late Lyme					
rP100 + PepVF + rOspC-K	94.3	80.84–99.30	90.0	68.30–98.77	0.976
rVlsE + PepVF + rOspC-K	100	90.00–100.0	95.0	75.13–99.87	0.994
rVlsE + rP41 + rOspC-K	100	90.00–100.0	95.0	75.13–99.87	0.997
rVlsE + rDbpA + rOspC-K	100	90.00–100.0	100	83.16–100.0	1.000
POC: Early + Late Lyme					
rP100 + PepVF + rOspC-K	88.5	69.74–95.19	90.0	68.30–98.77	0.844
rVlsE + PepVF + rOspC-K	94.3	80.84–99.30	75.0	50.90–91.34	0.932
rVlsE + rP41 + rOspC-K	71.4	53.70–85.36	75.0	50.90–91.34	0.807
rVlsE + rDbpA + rOspC-K	82.9	66.35–93.44	80.0	56.34–94.27	0.844

Abbreviations: CI, confidence interval; AUC, area under the curve.
