# Supplementary material for: Microfluidics-based point-of-care test for serodiagnosis of Lyme Disease
Source: Sci Rep. 2016 Oct 11;6:35069. doi: 10.1038/srep35069 (PMC5057150; doi:10.1038/srep35069)
Supplement: Supplementary Information [file srep35069-s1.pdf]

SUPPLEMENTARY INFORMATION

Microfluidics-based point-of-care test for serodiagnosis of Lyme Disease

Samiksha Nayak[1†], Archana Sridhara[1†], Rita Melo[2], Luciana Richer[3], Natalie H. Chee[1], Jiyoon Kim[1], Vincent Linder[4], David Steinmiller[4], Samuel K. Sia[1]* and Maria Gomes-Solecki[2,3,]*

[1] Department of Biomedical Engineering, Columbia University, 351 Engineering Terrace, 1210 Amsterdam Avenue, New York, NY 10027, USA.

[2] Department of Microbiology, Immunology and Biochemistry, University of Tennessee Health Science Center, 858 Madison Ave, Memphis, TN, 38163, USA

[3] Immuno Technologies Inc, 20 S Dudley St, Memphis TN 38103

[4] OPKO Diagnostics, LLC, 4 Constitution Way, Suite E, Woburn, MA, USA

[†] These authors contributed equally to this work.

[*] Correspondence to Maria Gomes-Solecki (mgomesso@uthsc.edu) and Samuel K. Sia (ss2735@columbia.edu)

Table S1. Characterization of Lyme-positive serum panel: clinical presentation and C6 ELISA/Western Blot data.

		Serological Tests		Reference Tests	
**Disease Stage**	**Sample**	**CDC C6 ELISA**	**CDC IgG WB**	**Bb Culture**	**Clinical Presentation**
Early Lyme	91-0521	Pos 2.53	Pos	NA	Early Disseminated, EM, Bells palsy
	91-0865	Pos 6.50	Pos	NA	Early Disseminated, EM, Bells palsy
	91-1350	Pos 1.97	**Neg**	Pos	Early Localized, EM
	91-1847	Pos 4.77	**Neg**	pos	Early Localized, EM
	91-1351	Pos 3.68	Pos	Pos	Early Localized, EM
	91-1842	Pos 4.54	Pos	Pos	Early Localized, EM
	91-1348	Pos 6.50	Pos	Pos	Early Localized, EM
	91-1347	Pos 4.51	**Neg**	Pos	Early Localized, EM
	91-1846	Pos 1.29	**Neg**	Pos	Early Localized, EM
	91-1458	**Neg 0.31**	**Neg**	Pos	Early Localized, EM
	90-2668	Pos 6.50	Pos	NA	Early Disseminated, Bells palsy
	91-1349	Pos 3.01	**Neg**	Pos	Early
	92-1982	Pos 1.31	**Neg**	Pos	Early Disseminated, EM
	91-1353	Pos 2.04	**Neg**	Pos	Early Localized, EM
	91-1844	Pos 5.62	Pos	Pos	Early Localized, EM
	91-1354	**Neg 0.57**	**Neg**	Pos	Early Localized, EM
	91-1845	**Neg 0.50**	**Neg**	Pos	Early Localized, EM
	91-1841	**Neg 0.61**	**Neg**	Pos	Early Localized, EM
	92-1941	Pos 1.53	**Neg**	Pos	Early Disseminated, EM
	90-2631	**Neg 0.25**	**Neg**	Pos	Early Disseminated, EM
	92-1682	Pos 1.33	**Neg**	Pos	Early Localized, EM
	91-1352	Pos 1.75	**Neg**	Pos	Early Localized, EM
	91-1222	Pos 5.77	**Neg**	Pos	Early Localized, EM
	91-1843	Pos 1.68	**Neg**	Pos	Early Localized, EM
	93-1414	Pos 6.18	Pos	Pos	Early Localized, EM
Late Lyme	91-0531	Pos 6.50	Pos	NA	Late, Arthritis, sinovitis
	91-0532	Pos 6.50	Pos	NA	Late Disseminated, arthralgies
	90-2111	Pos 6.07	Pos	NA	Late Disseminated, arthralgies
	91-0943	Pos 6.50	Pos	NA	Late Disseminated, arthralgies
	91-0794	Pos 2.73	Pos	NA	Late Disseminated, arthralgies
	91-0544	Pos 6.50	Pos	NA	Late Disseminated, arthralgies
	94-0357	Pos 2.69	Pos	Pos	Late Disseminated, Neuroborreliosis
	92-0057	Pos 6.50	Pos	Pos	Late Disseminated, arthralgies
	90-2436	Pos 1.75	Pos	NA	Late Disseminated, arthralgies
	91-0533	Pos 6.50	Pos	NA	Late Disseminated, arthralgies

Abbreviations: CI, confidence interval; Pos, positive; Neg, negative, NA, not applicable (not conducted); EM, Erythema migrans.

Bolded values are dissimilar serological test results from C6 ELISA or IgG Western Blot tests. Clinical classification descriptions are as follows: Erythema migrans, "bull's eye" rash; Arthralgies, joint pain; Sinovitis, inflammation of synovial membrane; Bells palsy, facial paralysis.

Table S2. Performance of two ELISA cutoffs for diagnosing Lyme disease (at any stage).

ELISA Performance with Cutoff Method 1 (High Specificity): Early + Late Lyme						
	**Sensitivity (%)**	**CI (95%)**	**Specificity (%)**	**CI (95%)**	**Cutoff**	**AUC**
**rP100**	42.9	26.32 - 60.65	100.0	88.78 - 100.0	0.250	0.971
**rBmpA**	31.4	16.85 - 49.29	95.0	75.13 - 99.87	0.296	0.936
**rOspA**	22.9	10.42 - 40.14	95.0	75.13 - 99.87	0.128	0.784
**rP41**	68.6	50.71 - 83.15	95.0	75.13 - 99.87	0.310	0.967
**rDpbA**	42.9	26.32 - 60.65	100.0	83.16 - 100.0	0.190	1.000
**rDbpB**	34.3	19.13 - 52.21	100.0	83.16 - 100.0	0.194	0.981
**rOspB**	20.0	8.44 - 36.94	100.0	83.16 - 100.0	0.154	0.714
**rOspC-K**	34.3	19.13 - 52.21	93.6	78.58 - 99.21	0.408	0.976
**rOspC-B**	54.3	36.65 - 71.17	100.0	88.43 - 100.0	0.444	0.996
**pepBBK07**	28.6	14.64 - 46.30	100.0	83.16 - 100.0	0.319	0.989
**rVlsE**	82.9	66.35 - 93.44	100.0	83.16 - 100.0	0.303	0.993
**PepVF**	77.1	59.86 - 89.58	95.0	75.13 - 99.87	0.124	0.961

ELISA Performance with Cutoff Method 2 (High Sensitivity): Early + Late Lyme						
	**Sensitivity (%)**	**CI (95%)**	**Specificity (%)**	**CI (95%)**	**Cutoff**	**AUC**
**rP100**	97.1	85.08 - 99.93	75.0	50.90 - 91.34	0.090	0.971
**rBmpA**	94.3	80.84 - 99.30	80.0	56.34 - 94.27	0.128	0.936
**rOspA**	71.4	53.70 - 85.36	70.0	45.72 - 88.11	0.070	0.784
**rP41**	100.0	90.00 - 100.0	95.0	75.13 - 99.87	0.157	0.967
**rDpbA**	100.0	90.00 - 100.0	100.0	83.16 - 100.0	0.064	1.000
**rDbpB**	100.0	90.00 - 100.0	95.0	75.13 - 99.87	0.060	0.981
**rOspB**	74.3	56.74 - 87.51	70.0	45.72 - 88.11	0.071	0.714
**rOspC-K**	94.3	80.84 - 99.30	95.0	75.13 - 99.87	0.106	0.976
**rOspC-B**	100.0	90.00 - 100.0	95.0	75.13 - 99.87	0.188	0.996
**pepBBK07**	100.0	90.00 - 100.0	95.0	75.13 - 99.87	0.103	0.989
**rVlsE**	100.0	90.00 - 100.0	95.0	75.13 - 99.87	0.158	0.993
**PepVF**	100.0	90.00 - 100.0	85.0	62.11 - 96.79	0.057	0.961

Abbreviations: CI, confidence interval; AUC, area under the curve.

Table S3. Results of ELISA screening using 12 antigens, segmented by Early-Lyme and Late-Lyme samples.

Table S3A

ELISA: Early Lyme					
	**Sensitivity (%)**	**CI (95%)**	**Specificity (%)**	**CI (95%)**	**AUC**
**rP100**	96	79.65 - 99.90	75	50.90 - 91.34	0.904
**rBmpA**	92	73.97 - 99.02	80	56.34 - 94.27	0.928
**rOspA**	68	46.50 - 85.05	70	45.72 - 88.11	0.755
**rP41**	100	86.28 - 100.0	95	75.13 - 99.87	0.964
**rDpbA**	100	86.28 - 100.0	100	83.16 - 100.0	1.000
**rDbpB**	100	86.28 - 100.0	95	75.13 - 99.87	0.984
**rOspB**	68	46.50 - 85.05	70	45.72 - 88.11	0.685
**rOspC-K**	96	79.65 - 99.90	95	75.13 - 99.87	0.983
**rOspC-B**	100	86.28 - 100.0	90	68.30 - 98.77	0.998
**pepBBK07**	100	86.28 - 100.0	95	75.13 - 99.87	0.988
**rVlsE**	100	86.28 - 100.0	95	75.13 - 99.87	0.990
**PepVF**	100	86.28 - 100.0	85	62.11 - 96.79	0.960

Table S3B

ELISA: Late Lyme					
	**Sensitivity (%)**	**CI (95%)**	**Specificity (%)**	**CI (95%)**	**AUC**
**rP100**	100	69.15 - 100.0	65	40.78 - 84.61	0.960
**rBmpA**	100	69.15 - 100.0	75	50.90 - 91.34	0.955
**rOspA**	80	44.39 - 97.48	65	40.78 - 84.61	0.855
**rP41**	100	71.51 - 100.0	90	68.30 - 98.77	0.973
**rDpbA**	100	69.15 - 100.0	95	75.13 - 99.87	1.000
**rDbpB**	100	69.15 - 100.0	90	68.30 - 98.77	0.975
**rOspB**	90	55.50 - 99.75	65	40.78 - 84.61	0.785
**rOspC-K**	90	55.50 - 99.75	90	68.30 - 98.77	0.960
**rOspC-B**	100	69.15 - 100.0	95	75.13 - 99.87	0.990
**pepBBK07**	100	69.15 - 100.0	90	68.30 - 98.77	0.990
**rVlsE**	100	69.15 - 100.0	90	68.30 - 98.77	1.000
**PepVF**	100	69.15 - 100.0	80	56.34 - 94.27	0.965

Abbreviations: CI, confidence interval; AUC, area under the curve.

Table S4. Performance of mChip-Ld screening using 8 antigens, for diagnosing Lyme disease at any stage.

	POC: Early + Late Lyme				
	**Sensitivity (%)**	**CI (95%)**	**Specificity (%)**	**CI (95%)**	**AUC**
**rP100**	68.6	50.71 - 83.15	70.0	45.72 - 88.11	0.699
**rP41**	62.9	44.92 - 78.53	44.0	24.40 - 65.07	0.579
**rDbpA**	51.4	33.99 - 68.62	80.0	56.34 - 94.27	0.650
**pepBBK07**	51.4	33.99 - 68.62	50.0	27.20 - 72.80	0.515
**rOspC-K**	68.6	50.71 - 83.15	92.3	74.87 - 99.05	0.778
**rOspC-B**	62.9	44.92 - 78.53	48.0	27.80 - 68.69	0.581
**rVlsE**	40.0	23.87 - 57.89	84.0	63.92 - 95.46	0.714
**PepVF**	94.3	80.84 - 99.30	73.1	44.33 - 82.79	0.934

Abbreviations: CI, confidence interval; AUC, area under the curve.

Table S5. Results of mChip-Ld screening using 8 antigens, segmented by Early-Lyme and Late-Lyme samples.

Table S5A

POC: Early Lyme					
	Sensitivity (%	CI (95%)	Specificity (%)	CI (95%)	AUC
**rP100**	68.0	46.50 - 85.05	70.0	45.72 - 88.11	0.680
**rP41**	60.0	38.67 - 78.87	56.0	34.93 - 75.60	0.643
**rDbpA**	56.0	34.93 - 75.60	80.0	56.34 - 94.27	0.621
**pepBBK07**	56.0	34.93 - 75.60	65.0	40.78 - 84.61	0.560
**rOspC-K**	84.0	63.92 - 95.46	92.3	74.87 - 99.05	0.877
**rOspC-B**	76.0	54.87 - 90.64	56.0	34.93 - 75.60	0.717
**rVlsE**	68.0	46.50 - 85.05	64.0	42.52 - 82.03	0.682
**PepVF**	92.0	73.97 - 99.02	73.1	52.21 - 88.43	0.914

Table S5B

POC: Late Lyme					
	Sensitivity (%)	CI (95%)	Specificity (%)	CI (95%)	AUC
**rP100**	70.0	34.75 - 93.33	70.0	45.72 - 88.11	0.745
**rP41**	60.0	26.24 - 87.84	64.0	42.52 - 82.03	0.582
**rDbpA**	60.0	26.24 - 87.84	95.0	75.13 - 99.87	0.723
**pepBBK07**	70.0	34.75 - 93.33	70.0	45.72 - 88.11	0.703
**rOspC-K**	40.0	12.16 - 73.76	96.2	80.36 - 99.90	0.529
**rOspC-B**	90.0	55.50 - 99.75	64.0	42.52 - 82.03	0.758
**rVlsE**	70.0	34.75 - 93.33	100.0	86.28 - 100.0	0.794
**PepVF**	100.0	69.15 - 100.0	96.2	80.36 - 99.90	0.985

Table S6. Description of Lyme antigens screened.

Family	Antigen	Name	Description
Membrane Proteins	OspA	Outer surface lipoprotein A	Protein in borrelial outer surface membrane; expressed mainly by B.burgdorferi in ticks[1]
	OspB	Outer surface lipoprotein B	Protein in borrelial outer surface membrane; expressed mainly by B.burgdorferi in ticks[1]
	OspC-K<br>OspC-B	Outer surface lipoprotein C, type K and type B	Protein in borrelial outer surface membrane; expressed by B.burgdorferi during transmission of spirochetes from ticks to mammals as well as in the vertebrate host in early infection [1, 5]
	p100	Membrane lipoprotein p100 (p93)	Immunodominant polypeptide
	BmpA	Basic membrane protein A (p39)	Borrelial outer membrane protein that binds to laminin in host's extracellular matrix; implicated as playing a role in some symptoms of Lyme disease[4]
	DpbA	Decorin binding protein A	Adhesin protein of B. burgdorferi that binds to decorin (a proteoglycan on surface of human cells); implicated in mediating tissue adherence of B. burgdorferi[2]
	DbpB	Decorin binding protein B	Adhesin protein of B. burgdorferi that binds to decorin (a proteoglycan on surface of human cells); implicated in mediating tissue adherence of B. burgdorferi[2]
	VlsE	Variable major protein-like sequence E	Surface exposed lipoprotein; belongs to a family of immunodominant variable major surface lipoproteins or VMPs that were involved in multiphasic antigenic variation in related Borrelia species[3]
Peptides	BBK07	Lipoprotein BBK07	Peptide isolated from a surface exposed lipoprotein
	PepVF	Peptide VF	Synthetic peptide isolated from a conserved region of VlsE and a fragment of flaB (p41)
Flagellar protein	flaB (p41)	Flagellin B, p41	Protein found in borrelial flagella

Sources:

[1] Pal, Utpal, et al. "OspC facilitates Borrelia burgdorferi invasion of Ixodes scapularis salivary glands." *The Journal of clinical investigation* 113.2 (2004): 220-230.

[2] Guo BP, Brown EL, Dorward DW, Rosenberg LC, Hook M. Decorin-binding adhesins from *Borrelia burgdorferi. Mol. Microbiol.* 1998;30:711–723.

[3] Zückert, Wolfram R. "A call to order at the spirochaetal host–pathogen interface." *Molecular microbiology* 89.2 (2013): 207-211.

[4] Verma, Ashutosh, et al. "Borrelia burgdorferi BmpA is a laminin-binding protein." *Infection and immunity* 77.11 (2009): 4940-4946.

[5] Coleman, Adam S., and Utpal Pal. "BBK07, a dominant in vivo antigen of Borrelia burgdorferi, is a potential marker for serodiagnosis of Lyme disease."Clinical and Vaccine Immunology 16.11 (2009): 1569-1575
